# From the bench to the bedside: Medulloblastoma subtypes, cholinergic neuron function in the nucleus accumbens, and mechanisms of chronic neuropathic pain

**DOI:** 10.4103/2152-7806.76718

**Published:** 2011-02-14

**Authors:** Jason S. Hauptman

**Affiliations:** Intellectual and Developmental Disabilities Center and Department of Neurosurgery, Geffen School of Medicine at UCLA, Los Angeles, CA, USA

## UNCOVERING THE DIVERGENT ORIGINS OF MEDULLOBLASTOMAs

Article: Subtypes of Medulloblastoma have Distinct Developmental Origins. Gibson P, Tong Y, Robinson G, Thompson MC, Currle DS, Eden C, Kranenburg TA, Hogg T, Poppleton H, Martin J, Finkelstein D, Pounds S, Weiss A, Patay Z, Scoggins M, Ogg R, Pei Y, Yang ZJ, Brun S, Lee Y, Zindy F, Lindsey JC, Taketo MM, Boop FA, Sanford RA, Gajjar A, Clifford SC, Roussel MF, McKinnon PJ, Gutmann DH, Ellison DW, Wechsler-Reya R, Gilbertson RJ.

Nature. 2010 Dec 23;468(7327):1095-9. Epub 2010 Dec 8.

PMID: 21150899 [PubMed - indexed for MEDLINE]

### Key Points

- Medulloblastomas, the most common malignant brain tumors in children, are composed of groups of tumors with different molecular origins. As such, medulloblastomas can be separated into subtypes based on the molecular pathways that are altered in them. Two of these subtypes include the SHH-subtype (in which sonic hedgehog signaling, a growth pathway, is altered) and WNT-subtype (in which a different growth pathway, the WNT pathway, is altered).
- Medulloblastomas behave differently based on their subtype. SHH-subtypes tend to present earlier, have worse prognoses, and are thought to arise from granule neuron precursor cells of the cerebellum and upper rhombic lip (these are primarily cerebellar tumors).
- WNT-subtypes tend to present later, have better prognoses, and appear to occur in the lower rhombic lip and dorsal brainstem. Unlike SHH-subtype medulloblastomas, WNT-subtype medulloblastomas actually arise within the dorsal brainstem and not in the cerebellum proper.
- In this paper, the authors develop a mouse model of the WNT-subtype medulloblastoma that is very similar to the human entity.
- Recognizing the distinct molecular origins of medulloblastoma subtypes may have a profound influence on diagnosis, prognosis, and therapy.

Medulloblastomas, the most common malignant brain tumors of childhood, are not uniform tumors. They have heterogeneous clinical characteristics as well as different molecular etiologies. Two distinct medulloblastoma subtypes have been differentiated based on molecular markers: tumors in which sonic hedgehog (SHH) signaling is dysregulated and tumors in which a different signaling pathway, driven by the transcription factor WNT, is disrupted. SHH-subtype medulloblastomas tend to have a poorer prognosis, occur in younger children, and arise from granule neuron precursor cells of the cerebellum. While it known that WNT-subtype medulloblastomas arise in older children and have an overall better prognosis, less is understood about their origin. In this study, investigators uncover more information regarding the divergent molecular origins of medulloblastomas. This important distinction may have profound implications on medulloblastoma prognosis and treatment.

First, the authors used databases that show the developmental expression of genes associated with both types of medulloblastomas to determine if there are any anatomic differences between the two. They found that genes associated with the SHH-subtype tended to be expressed in the cerebellum and upper rhombic lip (the rhombic lip is a thickened area of the developing hindbrain thought to contain the granule neuron precursor cells), while genes associated with the WNT-subtype tended to be expressed in the lower rhombic lip. This difference in anatomical preference was confirmed by examining medulloblastomas resected from children—SHH-subtype tumors were located within the cerebellum while WNT-subtype tumors were in the fourth ventricle and dorsal brainstem.

Because WNT-subtype medulloblastomas are associated with mutations in a gene named *CTNNB1*, the authors generated mice where this *CTNNB1* gene would be deleted in certain hindbrain cell populations. They found that *CTNNB1* deletion had no impact on the proliferation of ventricular zone cell or cerebellar granule cell precursor neurons (types of cells known to be affected in the SHH-subtype medulloblastoma). Instead, *CTNNB1*-deletion mice developed abnormal cell proliferation in the dorsal aspect of the brainstem adjacent to the cerebellum. Furthermore, using molecular techniques, the authors found that the *CTNNB1* mutation directly affects the ability of dorsal brainstem precursor cells to differentiate and migrate, such that they “stall” and collect within the dorsal brainstem. There is a suggestion that these cells might be precursors of mossy fibers (before they migrate to their final destination), but this is not certain.

In a final set of experiments, the authors sought to elicit brainstem tumor formation in *CTNNB1* mutants. By themselves, *CTNNB1* deletions resulted in collections of the dorsal brainstem cells but no tumor formation. When this deletion was paired with a concurrent *p53* loss (a well-known gene that, when mutated, causes uncontrolled cell growth), however, mice began to develop medulloblastomas. This suggests that WNT-subtype medulloblastomas may be the result of concurrent *CTNNB1* and *p53* loss. This was further validated when the authors found that the genetic “signatures” of these mouse tumors matched human WNT-subtype medulloblastomas.

In all, this is a critical assessment of the different molecular characteristics within the medulloblastoma population. The concept that these tumors may have divergent molecular origins is critical—it appears to associate with presentation and survival. Future diagnosis, prognostication, and therapeutic strategies will likely need to account for medulloblastoma subtype.

## THE ROLE OF CHOLINERGIC NEURONS IN THE NUCLEUS ACCUMBENS AND DRUG ADDICTION

Article: Cholinergic Interneurons Control Local Circuit Activity and Cocaine Conditioning. Ilana B. Witten, et al. Science 330, 1677 (2010);

DOI: 10.1126/science.1193771

### Key Points

- Cholinergic neurotransmission in the nucleus accumbens plays a role in models of drug addiction. The nucleus accumbens is made up of cholinergic (ChAT, ~1%) neurons and medium spiny neurons (MSNs, >95%).
- ChAT neuron activation results in inhibition of the majority of MSNs, while ChAT inhibition results in stimulation of the majority of MSNs. Cocaine directly stimulates ChAT neurons. Suppression of ChAT neurons results in a reduction to cocaine-induced conditioned place preference (a behavior correlate of cocaine-dependent reward behavior).
- ChAT neurons of the nucleus accumbens may be an appropriate target for interventional strategies treating drug addiction.

While cholinergic neurotransmission is widespread within the central nervous system, little is known about how the activity of cholinergic neurons affects behavior. Cholinergic neurons within the nucleus accumbens (an area of the ventral striatum implicated in the reward sytem), for instance, have been shown to have contradictory roles in the modulation of drug addiction. Cholinergic interneurons make up about 1% of the cells in the nucleus accumbens, with the remaining majority of cells being MSNs. Cholinergic neurons are thought to locally modulate the activity of MSNs within the nucleus accumbens. In this study, the authors use optogenetics (a method of using certain wavelengths of light to selectively activate or inhibit very specific groups of neurons) to assess the role of cholinergic neurons in the nucleus accumbens in the behavior of awake, active animals.

First, the researchers selectively expressed engineered proteins within the cholinergic (ChAT) neurons of the nucleus accumbens that very specifically either activate (ChR2) or inhibit (NpHR) the neuron when exposed to light from a fiberoptic probe. When ChAT neurons were stimulated with light, the result was an increase in GABA (inhibitory neurotransmitter) activity onto the MSNs. This shows that ChAT neuron activation leads to GABA release onto MSNs. This appeared to result in an overall reduction in MSN firing, though some MSNs actually increased firing (thought to be due to recurrent inhibitory collaterals between MSNs that are suppressed by ChAT activation; in other words, some MSNs had their inhibitory inputs suppressed). When the opposite experiment was conducted—that is, ChAT neurons were inhibited by light using the NpHR protein, firing of MSNs in the nucleus accumbens was markedly increased.

In the final set of experiments, the authors aimed to see what ChAT modulation would do to drug-related reward mechanisms. First, they applied *in vitro* cocaine to ventromedial ChAT neurons that were recorded from brain slices. The ChAT neurons were identified by filling them with a fluorescent protein. They found that cocaine application resulted in increased firing rates of ChAT neurons. They then shifted focus toward behavior in awake, active mice by looking at conditioned place preference (where a mouse learns to associate a particular environment with cocaine). They found that when ChAT neurons were silenced (using the NpHR protein), conditioned place preference was significantly decreased. Furthermore, when ChAT neurons were silenced in the absence of cocaine, no effects were seen. This is a particularly interesting set of findings, especially when considering alternative measures for treating addictive behaviors. If one was to consider targeting the nucleus accumbens for therapy, it is clear that the most important cellular targets are the ChAT cells.

## GETTING TO THE ROOT OF CHRONIC NEUROPATHIC PAIN

Article: Alleviating Neuropathic Pain Hypersensitivity by Inhibiting PKMζ in the Anterior Cingulate Cortex Xiang-Yao Li, *et al*. Science 330, 1400 (2010);

DOI: 10.1126/science.1191792

### Key Points

- Synaptic plasticity, the method by which the strength of synapses is changed in response to a given stimulus, is a way that a neuronal circuit “learns” how to respond to a given stimulus. PKMζ is a protein that is important for synaptic plasticity to occur.
- The anterior cingulate cortex (ACC) has been implicated in the development of chronic neuropathic pain syndromes. It turns out that following injury, PKMζ levels are elevated for ~3 days post-injury while activated PKMζ (p-PKMζ) levels remain consistently elevated, suggesting potential changes in synaptic plasticity.
- ZIP, a peptide that inhibits PKMζ, causes analgesia when infused into the ACC of mice following nerve injury.
- It appears that PKMζ-mediated chronic neuropathic pain is due to changes in glutamatergic neurotransmission at synapses of ACC neurons. In fact, enhanced gluatamtergic transmission following nerve injury is reversed by ZIP.
- This work is central to the understanding of the neural mechanisms of chronic pain, which appear to be a maladaptive form of synaptic plasticity. It is likely that modulation of the ACC may play a role in therapy for intractable chronic pain.

Synaptic plasticity, the method by which neurons “learn” and adapt responses to stimuli, has been implicated in the development of chronic pain syndromes. It has previously been noted that sensory nerve injuries, for example, trigger changes all along the sensory pathway from the nerve terminals themselves to the spinal cord, limbic structures, and cortex. Understanding these changes is critical to elucidating the mechanisms underlying pharmacologically resistant pain states and subsequently developing alternative therapies. One particular protein of interest is PKMζ, a protein that is known to play an important role in synaptic plasticity.

The authors began by examining PKMζ (unactivated) and p-PKMζ (activated) levels in the ACC, an area known to be involved in chronic pain states. They found that 3 days following nerve injury, both PKMζ and p-PKMζ levels were increased in the ACC, corresponding to allodynic behavior (pain response to something that would otherwise not normally be painful). By 7 days, PKMζ levels returned back to baseline while the activated (p-PKMζ) levels remained elevated. These changes in PKMζ and p-PKMζ were specific to the ACC such that levels in the hippocampus and spinal cord were not changed. They then found that this increase in PKMζ and p-PKMζ was dependent on another secondary messenger, calmodulin-stimulated adenylyl cyclase 1 (AC1). In fact, mice lacking AC1 did not experience allodynia or have detectable changes in PKMζ levels following nerve injury.

The authors then infused the ACC of intact animals with a PKMζ inhibitor, ZIP, to see what PKMζ inhibition would do following nerve injury. As expected, at both 3 and 7 days after nerve injury, ZIP reduced allodynia (an effect that was seen within 2 hours of injection and lasted for about 24 hours). Furthermore, this effect of ZIP was specific to the ACC—when it was injected elsewhere, no analgesic effects were noted. Similar ZIP-induced analgesia was seen in a model of nerve-injury related tonic pain.

In the next set of experiments, the authors worked to uncover the potential mechanism by which ZIP (and thus PKMζ) changes synaptic plasticity in the ACC. Using electrophysiology techniques in brain slices after nerve injury, they found that the likely mechanism by which PKMζ changes cortical synapses is by increasing the number of glutamate receptors (specifically, AMPA receptors). This, in essence, makes the cells more sensitized to excitatory stimuli.

In the final experiments, the authors used mice that expressed a green fluorescent protein (GFP) in ACC neurons that were activated by the nerve injury (they measured this activation by linking GFP to the gene *c-fos*, a gene known to be activated in sensory neurons following injury). They found that in the activated ACC neurons, there was enhanced excitatory synaptic transmission that was blocked with ZIP. This again confirmed the notion that the ACC neurons undergo a form of maladaptive synaptic plasticity, making them abnormally sensitive to excitatory stimuli. This fascinating set of experiments helps to shed light on the mechanisms by which chronic neuropathic pain occur. It also raises the question of ACC targeting for chronic pain disorders.

